# Environmental Behavior of 2,4,6-Trichlorophenol in the Sediment-Overlying Water System with the Presence of Tubificid Worms

**DOI:** 10.3390/toxics14040314

**Published:** 2026-04-07

**Authors:** Leyuan Zhang, Deming Dong, Xinyan Fu, Yu Zhao, Meihan Bao, Xiuyi Hua, Dapeng Liang, Haiyang Liu

**Affiliations:** Key Lab of Groundwater Resources and Environment of Ministry of Education, Key Lab of Water Resources and Aquatic Environment of Jilin Province, College of New Energy and Environment, Jilin University, Changchun 130012, China

**Keywords:** bioturbation, sediments, tubificid worms, 2,4,6-trichlorophenol, bacterial community

## Abstract

To investigate the influence of bioturbating organisms on the migration and degradation of chlorophenols in freshwater sediments, simulated experimental systems were established, with tubificid worms employed as the model bioturbator and 2,4,6-trichlorophenol (TCP) as a representative chlorophenol contaminant. The results showed that tubificid worms significantly promoted the removal of TCP in sediments, with this effect mainly concentrated in the surface sediment layer (0–2 cm) and limited impact on deeper sediment layers (2–6 cm). The removal efficiency was higher in the low-concentration TCP group than in the high-concentration group. TCP in the overlying water was predominantly in the dissolved phase, and the presence of tubificid worms reduced the TCP concentration in the aqueous phase, resulting in a greater amount of removal. The bioturbation of tubificid worms altered the physicochemical characteristics of the system, increasing the turbidity of the overlying water, decreasing its pH, elevating the redox potential across different sediment depths, and improving the organic matter conditions. Tubificid worms also modified the bacterial community structure in both the overlying water and the sediment. The core mechanism by which tubificid worms accelerate TCP removal is through promoting the migration of TCP from the sediment to the overlying water, while concurrently regulating the bacterial community structure in the overlying water to enhance the degradation capacity of chlorophenols in this layer. This highlights the important role of bioturbators in aquatic ecosystems, and ignoring their presence may lead to an erroneous underestimation of the system’s self-purification capacity.

## 1. Introduction

Sediments, as a key component of natural aquatic environments, serve not only as important reservoirs of organic matter and nutrients but also as major sinks and potential sources for various pollutants in water bodies [1]. Dissolved pollutants can be immobilized in sediments through adsorption, contaminated suspended particles may settle onto the sediment surface, and previously sequestered contaminants may be re-released into the overlying water due to changes in environmental conditions, forming a dynamic cycle of adsorption–settling–release [2]. It is noteworthy that organic matter within sediments is not static, it undergoes complex transformation and degradation processes driven by multiple factors such as microbial metabolism, shifts in redox conditions, and bioturbation by benthic organisms. These processes not only regulate the balance of sediment carbon pools but also indirectly influence the environmental fate of pollutants by altering the forms and bioavailability of organic matter [3].

Sediments simultaneously provide a vital habitat for diverse benthic organisms. In nature, marine and freshwater ecosystems host a variety of bioturbating organisms that serve as critical drivers of material cycling at the sediment–water interface. Current research predominantly focuses on marine ecosystems, with typical model organisms including *Nereis*, *Uca* spp., *Mytilus edulis*, and Holothuroidea [4,5,6,7]. In freshwater ecosystems, common examples include tubificid worms, *Chironomus*, and *Macrobrachium nipponense* [8,9,10,11]. Among these, tubificid worms are one of the most widely distributed bioturbators in freshwater systems and belong to the phylum Annelida, class Oligochaeta, and family Tubificidae. They inhabit various freshwater sediments globally, including rivers, lakes, wetlands, and reservoirs, acting as a crucial biological link connecting sediment and water column ecological processes. Owing to their exceptional tolerance to hypoxia and pollution, tubificid worms are frequently employed as model bioturbators in environmental research [12,13].

At the sediment–water interface in natural water bodies, the environmental behavior of pollutants, including their migration, transformation, and ultimate fate, is often significantly regulated by benthic organism activity. For water bodies with relatively quiescent bottom waters, considering only molecular diffusion and sediment resuspension triggered by extreme events like storms while ignoring the mediating effects of bioturbation, would likely lead to a significant underestimation of actual pollutant release and consequently large errors in environmental risk assessment. Previous studies have confirmed that tubificid worms can markedly alter the physicochemical characteristics of sediments through life activities such as respiration, feeding, excretion, and burrowing. On the one hand, they promote material transport and exchange between sediments and overlying water, accelerating the flux of pollutants across the interface [14,15]. On the other hand, they regulate the local concentrations and distributions of key components such as dissolved oxygen (DO), CO_2_, and organic matter, reshaping the sediment microenvironment and driving shifts in microbial community structure, thereby indirectly affecting the efficiency of pollutant and nutrient transformation [16,17]. Furthermore, benthic organisms can modulate sediment redox conditions by enhancing the transport of oxygen and other electron acceptors into reducing environments, influencing bacterial metabolic rates and stimulating organic matter mineralization [18,19]. Concurrently, by altering biogeochemical gradients and organic matter bioavailability, they regulate bacterial community structure, ultimately exerting a significant influence on the degradation processes of organic pollutants [20].

Chlorophenols are a class of highly toxic and carcinogenic aromatic compounds, representing typical organochlorine pollutants. Their stable aromatic ring structure and chlorine substituents confer strong persistence and recalcitrance to biodegradation in the environment [21,22]. Chlorophenols are widely used as industrial raw materials or intermediates and often enter the environment via industrial waste (e.g., from oil refining and coking) or as direct pollutants [23]. They are relatively water-soluble and have been detected in surface water, groundwater, sediments, and soil [24,25]. Studies indicate that the no-effect concentration values for various chlorophenols in soil range from 0.75 mg/kg to 3.42 mg/kg [26]. Chlorophenol pollutants readily adsorb to soil or sediment organic matter and can also volatilize into the atmosphere, spreading via dry and wet deposition. Lower chlorinated phenols (e.g., 2,4-dichlorophenol) are more susceptible to microbial degradation, whereas highly chlorinated phenols (e.g., pentachlorophenol, PCP) are more stable, with anaerobic degradation being the primary natural attenuation pathway, followed by photodegradation and chemical oxidation [27]. Chlorophenols can biomagnify through the food chain, accumulating in aquatic organisms such as fish and shellfish, ultimately posing threats to higher trophic levels and human health. Due to their carcinogenic, mutagenic, and cytotoxic properties, the World Health Organization (WHO) and the U.S. Environmental Protection Agency (USEPA) have listed them as potential human carcinogens and priority control pollutants [28]. 2,4,6-Trichlorophenol (TCP), a commonly encountered chlorophenol compound, is extensively used in the production of pesticides, herbicides, and wood preservatives [29]. It can enter the food chain via bioaccumulation, causing irreversible harm to humans and other animals, such as inducing lymphoma, leukemia, and liver cancer [30].

In natural aquatic environments, sediments are the primary sink for TCP and other chlorophenol pollutants. Their biodegradation is a key pathway for the removal of such contaminants, with the core process being dechlorination, primarily divided into reductive dechlorination and oxidative dechlorination. Reductive dechlorination involves chlorophenol compounds accepting electrons while losing chlorine substituents and releasing chloride ions, occurring mainly under anaerobic or anoxic conditions through microorganisms transferring electrons (as [H]) to chlorinated aromatics [31,32,33]. Under aerobic conditions, the biodegradation of chlorophenols is accomplished through a series of enzymes entering the electron transport chain, typically following a ring cleavage before a dechlorination mechanism. Ring cleavage and fission require oxygen to extract electrons from the benzene ring molecule [34]. Additionally, co-metabolic degradation is another important pathway for chlorophenols, with degradation rates influenced by factors such as temperature, oxygen availability, and microbial species and activity [35,36].

The presence of bioturbating organisms like tubificid worms may indirectly affect microbial community composition and activity by altering sediment redox conditions and organic matter bioavailability. Simultaneously, these worms might facilitate the migration of TCP from sediments to the overlying water, thereby regulating the degradation, transformation, and migration processes of TCP in sediments. However, the underlying regulatory mechanisms remain unclear. To date, research on the influence of bioturbators on organic pollutant degradation has primarily focused on petroleum hydrocarbons and polycyclic aromatic hydrocarbons, with fewer studies on chlorophenols. Examples include *Perinereis aibuhitensis* enhancing the removal of petroleum hydrocarbons [37], as well as *Nereis diversicolor* and *Hediste diversicolor* promoting the metabolism of pyrene [38]. Furthermore, related research has concentrated mostly on marine ecosystems and marine bioturbators, with studies in freshwater systems being relatively scarce.

Therefore, to investigate the impact of benthic bioturbators on the migration and transformation of chlorophenol pollutants, this study selected tubificid worms as the model bioturbator and 2,4,6-trichlorophenol (TCP) as the target pollutant. Through laboratory simulation experiments, combined with the analysis of physicochemical parameters and microbial communities, we systematically explored the effect of tubificid worms on TCP degradation in sediments and its underlying driving mechanisms. This research aims to provide theoretical support for a deeper understanding of the biogeochemical processes of pollutants at the freshwater sediment–water interface and to offer a scientific basis for the ecological remediation of contaminated sediments.

## 2. Materials and Methods

### 2.1. Experimental Materials

The sediment used in the experiment was collected from Nanhu Lake in Changchun, China (43°51′ N, 125°18′ E). Surface sediments at a depth of 3–20 cm were collected, sealed in clean plastic buckets, and transported back to the laboratory. The sediment samples were wet-sieved through a 20-mesh sieve (0.85 mm), which effectively removed coarse impurities such as stones, plant debris, and macrobenthos while retaining the fine-grained matrix (e.g., clay and silt particles) essential for the experiment to the greatest extent, thereby avoiding substantial loss of the main sediment components. After collection and pretreatment, the sediment was analyzed for its initial physicochemical properties. The characterization results were as follows: pH 7.23 ± 0.15, ORP 186 ± 12 mV, TOC content 1.92 ± 0.08%, total nitrogen (TN) 0.23 ± 0.02%, and total phosphorus (TP) 0.12 ± 0.01%. Particle composition was determined using a laser particle size analyzer, with clay (<0.002 mm), silt (0.002–0.05 mm), and sand (0.05–2 mm) accounting for 28.6%, 65.3%, and 6.1%, respectively, indicating a typical fine-grained muddy sediment. The sediment water content was 32.2 ± 1.5%. The processed sediment was then stored in a ventilated and shaded area for subsequent use. The tubificid worms used in the experiment were purchased from the Qingyifang Market in Changchun. Before the formal experiments, the worms were acclimated under laboratory conditions for 7 days to adapt to the experimental environment and to select individuals with stable growth conditions. Microscopic observation of the tubificid worm samples revealed that *Limnodrilus hoffmeisteri* was the dominant species.

### 2.2. Simulation Experiment of TCP Environmental Behavior

#### 2.2.1. Experimental Setup

Cylindrical glass jars with a diameter of 6.5 cm and a height of 12 cm were used for the experiment. Approximately 6 cm (290 g) of pre-treated sediment was filled into the bottom of each jar. Prior to use, the target pollutant TCP was added to the sediment at predetermined concentrations. After thorough homogenization, the sediment was equilibrated for 5 days to ensure an initially uniform distribution of TCP. In all TCP concentration treatments, the sediment in both the group with tubificid worms and the group without tubificid worms was from the same batch of homogenized sediment. Synthetic freshwater (96 mg·L^−1^ NaHCO_3_, 39.4 mg·L^−1^ CaSO_4_·2H_2_O, 60 mg·L^−1^ MgSO_4_·7H_2_O, 4 mg·L^−1^ KCl, pH = 7.5) [17] was slowly added along the inner wall of the jar using a siphon to a height of approximately 5 cm (120 mL volume). A micro-air pump connected to a porous aeration stone was used to gently aerate the overlying water, with the aeration rate controlled to avoid significant disturbance to the sediment surface. All jars were evenly arranged in a constant-temperature water bath equipped with a heater and circulation system to maintain a stable water temperature of 20 ± 3 °C throughout the experiment. Prior to the introduction of tubificid worms, a quantitative screening was performed. Worms in batches of 10, 20, 40, 70, and 100 individuals with consistent growth conditions were selected, surface moisture was removed, and they were weighed to establish a linear relationship between worm weight and number for standardized quantification. The worm density in the experiment was set at 30,000 ind/m^2^, which aligns with typical natural densities of tubificid worms in freshwater sediments [39]. The experiment commenced immediately after system setup and lasted for 28 days.

#### 2.2.2. Experimental Procedures

Three pollutant concentration gradients were established: (1) Control group without TCP (CK^+^ and CK^−^ groups); (2) Low-concentration TCP group (L^+^ and L^−^ groups), with an initial TCP concentration of 2.48 ± 0.08 mg/kg; (3) High-concentration TCP group (H^+^ and H^−^ groups), with an initial TCP concentration of 5.20 ± 0.07 mg/kg. The selected low and high concentrations are consistent with actual detected levels of chlorophenol pollutants in natural environments [26]. Each system included conditions with tubificid worms (+) and without worms (−). Each group was set up with 12 replicate glass jars, from which three jars were sampled at each of the time points (day 7, 14, 21, and 28).

During the experiment, at each sampling time point, 10 mL of overlying water was slowly withdrawn along the inner wall of the glass jar using a sterile syringe, and turbidity, pH, and oxidation-reduction potential were measured on site. After measurement, the water sample was gently reintroduced along the jar wall to minimize disturbance to the system. Sediment sampling was conducted on days 7, 14, 21, and 28. The overlying water was first completely removed, and then sediment was collected in layers using a spatula, with layer depths of 0–1 cm, 1–2 cm, 2–4 cm, and 4–6 cm. The jars used for sampling were discarded and not used for further cultivation. Sediment samples from each layer were collected from two replicate jars, with two subsamples taken from each jar, yielding a total of four replicate samples per layer per group. At the end of the experiment (day 28), oxidation-reduction potential (ORP) was measured in six sediment layers (each 1 cm thick, covering 0–6 cm). Additionally, surface (0–2 cm) and subsurface (2–4 cm) sediment samples were collected for total organic carbon (TOC) analysis and bacterial community structure analysis.

### 2.3. Analytical Methods

The extraction of TCP from sediment employs methanol ultrasonic extraction method [40]. The overlying water sample was separated into particulate matter and filtrate through suction filtration. After extracting the particulate matter, the filtrate and extract solution were separately concentrated for testing. Detailed procedures and testing methods are provided in Text S1. Liquid chromatography–mass spectrometry (LC-MS; HPLC: Waters 2695; MS: Waters ZQ2000, Waters Corporation, Milford, MA, USA) was used to qualitatively identify intermediate products generated during the degradation of 2,4,6-trichlorophenol. Turbidity of the overlying water was measured with a turbidimeter (DR900, Hach Company, Loveland, CO, USA). Redox potential in both overlying water and sediment was determined using an ORP meter (FJA-6, Nanjing Chuandi Instrument Equipment Co., Ltd., Nanjing, China). pH was measured with a precision pH meter (PHS-3C, Shanghai Yidian Scientific Instrument Co., Ltd., Shanghai, China). Total organic carbon (TOC) in sediment was analyzed using a TOC analyzer (TOC-L with SSM-5000A, Shimadzu Corporation, Kyoto, Japan). Bacterial communities in sediment and overlying water were analyzed via 16S rRNA gene sequencing. The V3–V4 hypervariable region of the bacterial 16S rRNA gene was selected for PCR amplification with barcoded universal primers 338F and 806R, and the qualified amplicons were sequenced on an Illumina NovaSeq high-throughput sequencing platform. DNA extraction, PCR amplification, sequencing, and subsequent community structure analysis and functional prediction were conducted by Shanghai Paiseek Biotechnology Co., Ltd. (Shanghai, China). Specific experimental procedures and detailed data analysis methods are provided in Text S2. One-way analysis of variance (ANOVA) followed by Duncan’s multiple range test was used to determine significant differences between treatment groups at a significance level of *p* < 0.05. Statistical analyses were conducted using SPSS 26.0 (IBM Corp., Armonk, NY, USA). Graphs were constructed using Origin 2023 (OriginLab Corp., Northampton, MA, USA).

The core measurement parameters of this study include: (1) Pollutant indicators: Concentrations of dissolved/particulate TCP in sediment and overlying water. The core methods are described in Section 2.3 of the main text, and the extraction and detection details are provided in Supplementary Materials Text S1. (2) Physicochemical parameters: pH, ORP, turbidity of overlying water, ORP and TOC of sediments. The core methods are described in Section 2.3 of the main text, and the measurement structure is detailed in Supplementary Materials Text S3–S7. (3) Microbial indicators: The core amplification and sequencing information is described in Section 2.3 of the main text, and the detailed process and data analysis are detailed in Supplementary Materials Text S2.

## 3. Results and Discussion

### 3.1. Migration and Degradation of TCP

#### 3.1.1. Changes in TCP Concentration in Sediments

**Figure 1:**
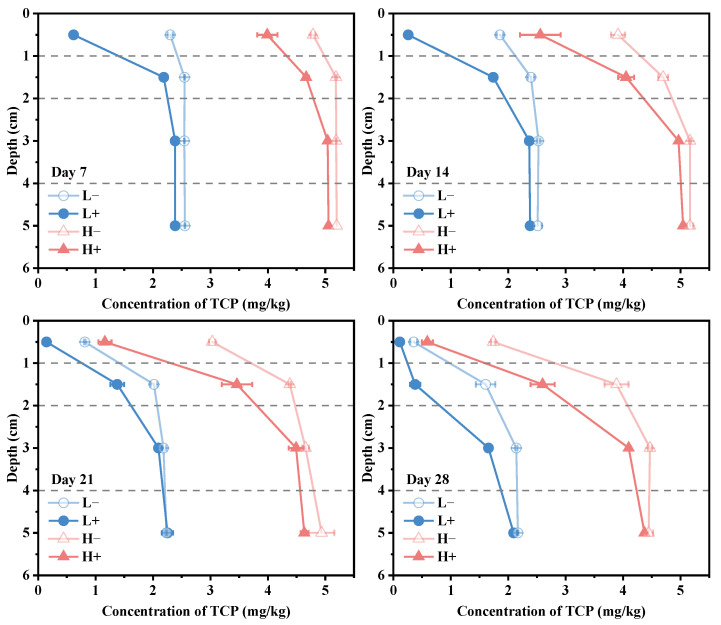
TCP concentration at different sediment depths (0–1, 1–2, 2–4, 4–6 cm) over time (days 7, 14, 21, and 28) in the four groups.

**Figure 2:**
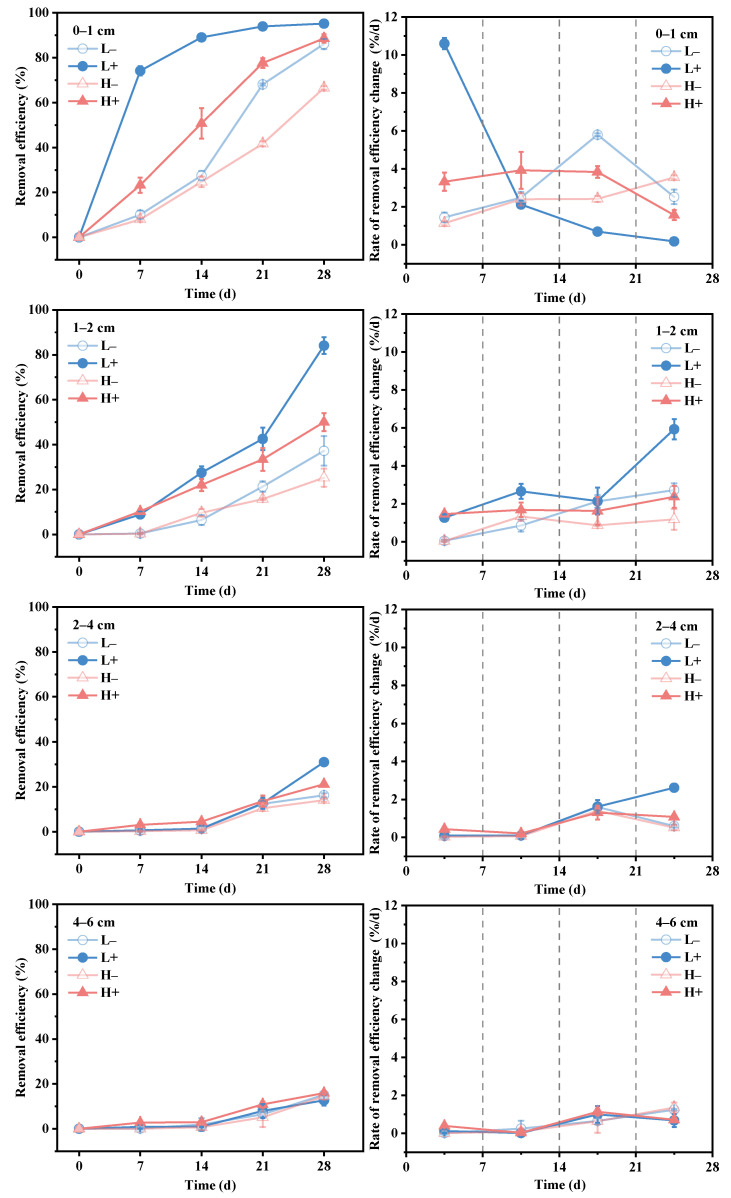
TCP removal efficiency and rate of its change over time at different sediment depths (0–1, 1–2, 2–4, 4–6 cm) in the four groups.

Figure 1 illustrates the distribution of TCP concentrations in sediments at different depths on days 7, 14, 21, and 28 of the experiment. Figure 2 presents the removal efficiency and removal rates of TCP across sediment layers. The variations in concentration, removal efficiency, and removal rates at each depth are summarized as follows:

In the surface sediment layer (0–1 cm), the enhancement of TCP removal by tubificid worms was most pronounced. On day 28, the TCP concentration in the L^+^ group (0.11 mg/kg) was 69.4% lower than that in the L^−^ group (0.36 mg/kg), while the concentration in the H^+^ group (0.59 mg/kg) was 66.1% lower than that in the H^−^ group (1.74 mg/kg). This indicates that tubificid worms significantly accelerated the reduction of TCP in the surface layer. In the L^−^ group, the removal rate followed a typical “S-shaped curve” (initial adaptation, intermediate acceleration, and final slowdown) [41]. In contrast, given the higher initial concentration in the H^−^ group, it is inferred that the microbial adaptation period—the stage during which indigenous degrading bacteria acclimate to high TCP stress, achieve metabolic activation, and undergo population proliferation—was prolonged, which has resulted in a gradual increase in the removal rate over time. Comparatively, the removal rate in the L^+^ group peaked at 10.6% on day 7, with a removal efficiency of 74.2% by day 7 (64.1% higher than that of the L^−^ group). The removal rate subsequently declined. In the H^+^ group, the removal efficiency increased linearly from days 0 to 21 (with rates maintained between 3.3% and 3.9%), followed by a decline after day 21. These results demonstrate that the presence of tubificid worms significantly enhanced the removal efficiency of TCP in the surface layer, likely due to the stimulation of microbial community growth and activity in the sediment.

In the subsurface sediment layer (1–2 cm), the promoting effect of tubificid worms was diminished but remained significant. On day 28, the TCP concentration in the L^+^ group (0.38 mg/kg) was 76.4% lower than that in the L^−^ group (1.61 mg/kg), while the concentration in the H^+^ group (2.60 mg/kg) was 33.2% lower than that in the H^−^ group (3.89 mg/kg). These differences between groups with and without worms were statistically significant (*p* < 0.05). Although the enhancement effect on TCP degradation was weaker than in the surface layer, significant differences were still observed. The removal rates across all groups remained at low levels (0.06–5.9%). However, the removal rate in the L^+^ group increased sharply to 5.9% during the period from day 21 to day 28, which may indicate that the microbial adaptation period of indigenous degrading bacteria in this group had been completed by the middle-to-late stage of the experiment, with bacterial activity elevated, and the degradation rate is likely to accelerate thereafter.

In the deeper sediment layers (2–4 cm and 4–6 cm), the promoting effect of tubificid worms was limited. In the 2–4 cm layer, significant differences between groups with and without tubificid worms were observed only on day 28. The TCP concentration in the L^+^ group (1.66 mg/kg) was 22.4% lower than that in the L^−^ group (2.14 mg/kg), while the concentration in the H^+^ group (4.10 mg/kg) was 8.3% lower than that in the H^−^ group (4.47 mg/kg). Removal rates across all groups remained low (0.07–2.6%). In the 4–6 cm layer, no significant differences in TCP concentrations were observed between groups with and without tubificid worms (*p* > 0.05). Removal rates across all groups remained at low levels (0–1.4%). These results indicate that the bioturbation by tubificid worms primarily promoted TCP removal in the surface sediment layers, with limited impact on deeper layers.

In summary, the TCP concentrations in all treatment groups decreased over time. However, the reduction rates in groups with tubificid worms (L^+^, H^+^) were significantly higher than those in groups without worms (L^−^, H^−^). This difference was particularly evident in the surface sediment layers (0–2 cm). Overall, the removal efficiency was higher in the low-concentration groups compared to the high-concentration groups.

#### 3.1.2. Changes in Overlying Water TCP Concentration

**Figure 3:**
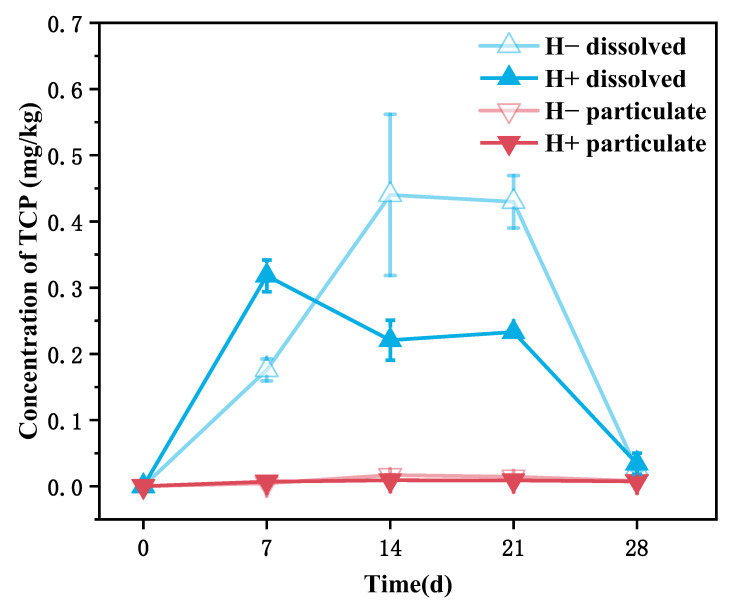
Changes in dissolved and particulate TCP concentrations in the overlying water with and without tubificid worms.

The variations in dissolved and particulate TCP concentrations (taking the high-concentration group as an example) are shown in Figure 3. In the H^−^ group, the dissolved TCP concentration initially increased and then gradually decreased, peaking at 0.59 mg/L on day 14. In contrast, the dissolved TCP concentration in the H^+^ group remained relatively stable, ranging between 0.16 mg/L and 0.22 mg/L from day 7 to day 21. A comparative analysis reveals that the dissolved TCP concentration in the H^−^ group was significantly higher than that in the H^+^ group, with differences of 0.30 mg/L, 0.41 mg/L, and 0.21 mg/L on days 7, 14, and 21, respectively. By day 28 of the experiment, the dissolved TCP concentrations in both groups had decreased to low levels of approximately 0.018–0.02 mg/L. Meanwhile, the particulate TCP concentrations in the overlying water of both groups remained consistently low throughout the experiment, ranging from 0.0052 mg/L to 0.012 mg/L. These results indicate that the presence of tubificid worms significantly reduced the accumulation of TCP in the overlying water. TCP mainly exists in dissolved form, with a relatively small proportion in particulate form.

#### 3.1.3. Phase Distribution and Degradation Contribution of TCP

**Figure 4:**
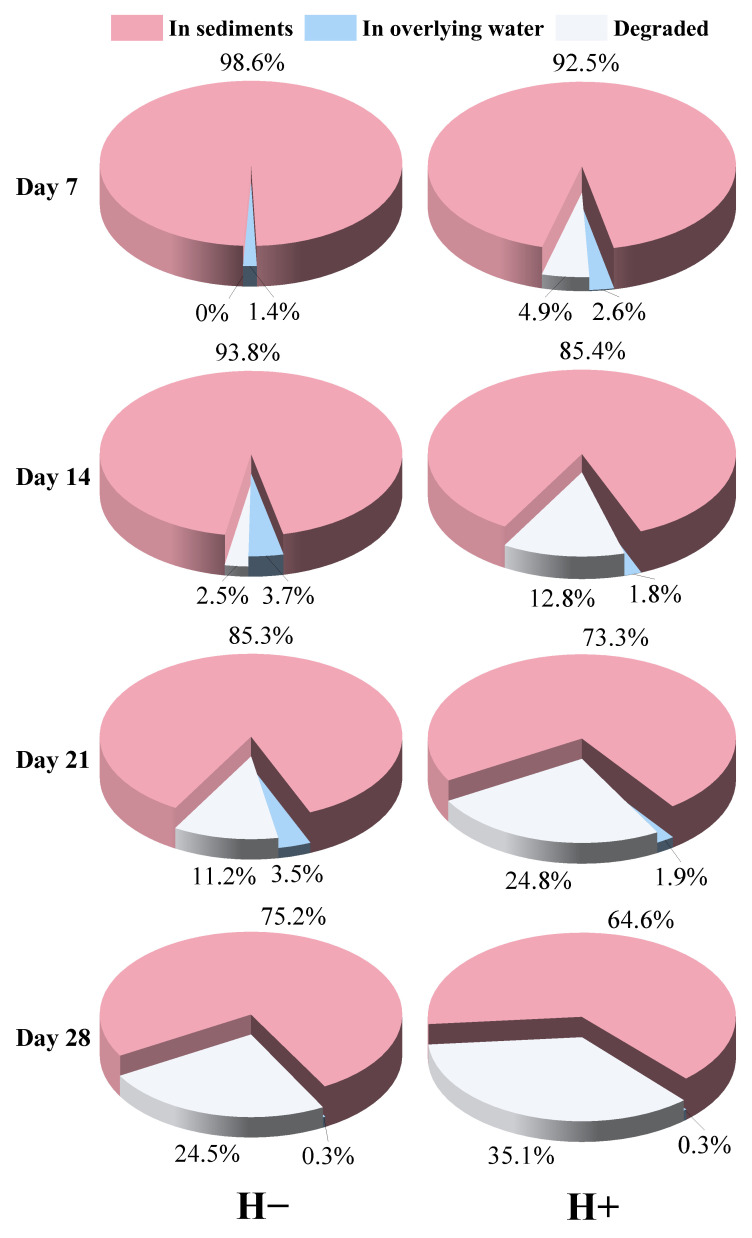
Phase distribution and degradation contribution of TCP in the overlying water–sediment system with and without tubificid worms.

The phase distribution and degradation contribution of TCP are shown in Figure 4. Given that borosilicate glass containers were used in the experiments, which exhibit negligible sorption of TCP, the sorption loss was not considered. Additionally, the biomass of tubificid worms accounts for a small fraction of the entire experimental system, and TCP accumulation in tubificid worm biomass is not a primary pathway for TCP removal. Therefore, this portion was grouped together with the degradation fraction for joint analysis. In the group without tubificids, nearly all of the TCP removed from the sediment had diffused into the overlying water by day 7. Over time, the extent of degradation gradually increased, yet TCP in the overlying water continued to account for a substantial proportion. This indicates that diffusion into the overlying water was one of the primary mechanisms responsible for the reduction of TCP in the sediment. In contrast, in the group with tubificids, the proportion of TCP in the overlying water remained consistently low, while degradation played a more significant role. Numerous studies have demonstrated that the bioturbation activity of tubificids continuously mixes sediment and overlying water, thereby accelerating the transport of TCP into the overlying water (Gkika et al., 2024; Zhang et al., 2024) [42,43]. This also explains the marked decrease in TCP content observed in the surface sediment layer (0–2 cm) during the early stage of the experiment. However, in the presence of tubificids, TCP concentration in the overlying water did not increase; in fact, it was significantly lower than that in the system without tubificids. This phenomenon may be attributed to changes in the composition and activity of the microbial community in the overlying water influenced by the tubificids.

### 3.2. Changes in Physicochemical Properties of Overlying Water and Sediments

Changes in the oxidation-reduction potential (ORP) of overlying water are presented in Text S3 and Figure S1. The group without TCP exhibited relatively minor fluctuations, decreasing from an initial value of 509.4 ± 3.1 mV to a minimum of 475.4 ± 5.3 mV. In contrast, the ORP in TCP-contaminated groups declined rapidly after day 7, with each decreasing by over 160 mV. The ORP of the low-concentration group gradually rebounded after day 14, while the high-concentration group gradually rebounded after day 19. The research results on sediment ORP are shown in Text S4 and Figure S2. The sediment ORP gradually decreased with increasing depth, and the ORP in the groups with tubificid worms was consistently higher than that in the groups without tubificid worms. Additionally, the ORP of TCP-contaminated sediments was lower than that of the control group, but there was no linear relationship between TCP concentration and ORP.

Changes in overlying water turbidity are provided in Text S5 and Figure S3. The presence of tubificid worms significantly increased the turbidity of overlying water, which was attributed to the bioturbation of tubificid worms that increased sediment porosity and continuously stirred the surface sediment, promoting the resuspension of particles [16]. This process may have facilitated the migration of TCP from sediments to the overlying water, which is consistent with the result of the decrease in TCP content in surface sediments (as discussed in Section 3.1.1).

Changes in overlying water pH are described in Text S6 and Figure S4. The pH variation in the groups without tubificid worms was relatively small, while the pH in the groups with tubificid worms was consistently lower than that in the system without tubificid worms, showing a gradual decrease and a trend towards stability. In the group with tubificid worms and TCP, the pH was consistently lower than that in the control group, and the higher the TCP concentration, the lower the pH. This may be due to the activation of acid-producing metabolic pathways by specific microorganisms during the degradation process of pollutants.

The total organic carbon (TOC) contents in the surface layer (0–2 cm) and deep layer (2–4 cm) of sediments in different groups are described in Text S7 and Figure S5. The results showed that the sediment TOC content in all groups ranged from 1.7% to 2.2%. In the group without TCP, tubificid worms increased the sediment TOC, while in the groups with TCP, the presence of tubificid worms decreased the sediment TOC content. There was no significant difference in sediment TOC among the three groups without tubificid worms.

### 3.3. Characteristics of Bacterial Community Changes

#### 3.3.1. Bacterial Community in the Overlying Water

**Figure 5:**
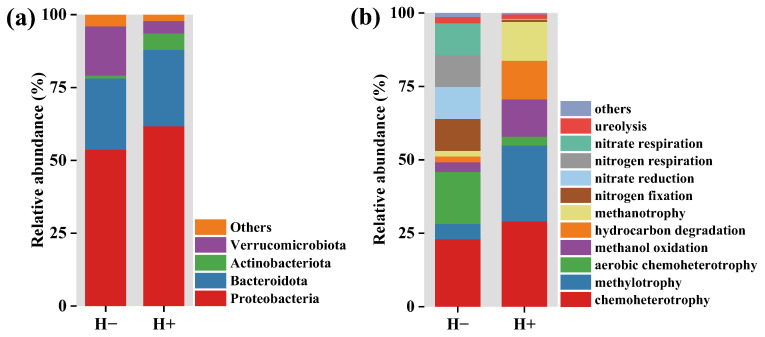
Relative abundance of bacteria in overlying water in systems with and without tubificid worms: (**a**) at phylum level and (**b**) of predicted functional groups.

To investigate the potential influence of bacteria on the reduction of TCP, the bacterial community and its potential functional groups were examined. For the overlying water, the H^−^ and H^+^ groups were selected as representative cases for bacterial community and functional analysis. Figure 5a illustrates the relative abundances of bacterial phyla in the overlying water on day 28. Four dominant phyla were identified: *Proteobacteria*, *Bacteroidota*, *Actinobacteriota*, and *Verrucomicrobiota*. Compared with the worm-free control group (H^−^), the relative abundances of Proteobacteria, Bacteroidota, and Actinobacteria in the overlying water of the tubificid worm-treated group (H^+^) were significantly increased by 8.1%, 1.7%, and 4.6%, respectively, while that of Verrucomicrobia was significantly decreased by 12.7% (*p* < 0.05).

The potential functional profiles of bacteria in the overlying water are shown in Figure 5b. Compared to the H^−^ group, the relative abundances of five functional groups in the H^+^ group were significantly elevated (*p* < 0.01): chemoheterotrophy, methylotroph, methanol oxidation, hydrocarbon degradation, and methanotrophy, with increases of 6.1%, 20.6%, 9.3%, 11.3%, and 11.3%, respectively. Bioturbation by tubificid worms promotes the release of methane from sediments [44], providing an abundant energy source for methanotrophic bacteria. The metabolic byproducts of methanotrophs, such as methanol and acetate, can serve as substrates for methylotrophs and methanol oxidizers, thereby driving the proliferation of these functional groups. Studies have indicated that methylotrophy and methanol oxidation are potential metabolic pathways for the degradation of chlorophenol compounds [45]. Meanwhile, hydrocarbon-degrading bacteria can metabolize alkanes and aromatic hydrocarbons through aerobic or facultative anaerobic pathways, which also contribute to the degradation of chlorophenol pollutants.

In summary, the presence of tubificid worms significantly enhanced the abundances of functional groups directly associated with chlorophenol degradation in the overlying water (methylotrophy, methanol oxidation, and hydrocarbon degradation). This represents a key microbial-driven mechanism underlying the enhanced TCP degradation efficiency in systems with tubificid worms.

#### 3.3.2. Bacterial Community in Sediment

**Figure 6:**
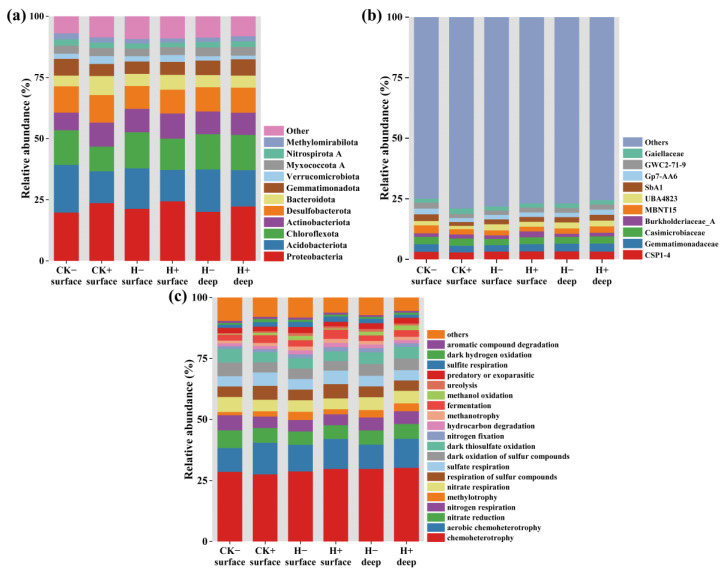
Bacterial abundance in surface (0–2 cm) and deep (2–4 cm) sediments of different groups (**a**) at the phylum level and (**b**) at the family level, as well as (**c**) of predicted functional groups.

Figure 6a illustrates the relative abundance distribution of sediment bacterial communities at the phylum level across treatment groups. The results indicate that each system contained 7–8 dominant phyla (defined as phyla with relative abundance > 5%). *Proteobacteria* emerged as the most dominant phylum in all groups, with relative abundances ranging from 20.1% to 25.1%, followed by *Acidobacteriota* (12.9–19.5%), *Chloroflexota* (10.0–14.5%), *Actinobacteriota* (7.2–9.4%), *Desulfobacterota* (9.2–11.2%), and *Gemmatimonadota* (4.9–6.8%). Notably, *Bacteroidota* was identified as a dominant phylum only in surface sediments of the control group with tubificid worms (CK^+^) and the high-concentration group with tubificid worms (H^+^), with relative abundances of 6.2–7.7%. The presence of tubificid worms altered the relative abundances of dominant phyla in sediments. *Proteobacteria* showed a significant increase in worm-present groups, with increments of 3.7% in the control group, 4.2% in surface sediments of experimental groups, and 1.5% in deeper sediments compared to worm-free groups. In contrast, the relative abundances of *Acidobacteriota* and *Chloroflexota* generally decreased in worm-present groups. Specifically, *Acidobacteriota* decreased by 6.6% in the control group, 3.1% in surface sediments, and 2.9% in deeper sediments. *Chloroflexota* decreased by 4.2%, 1.2%, and 0.4%, respectively. Pearson correlation analysis between bacterial relative abundances at the phylum level and system variables (with/without tubificid worms, sediment depth, presence/absence of contaminant) is presented in Figure S6. The results indicated no significant correlations (*p* > 0.05) between any bacterial phylum and the examined factors. *Bacteroidota* and *Firmicutes* showed positive correlations with worm presence, while most phyla exhibited negative correlations with sediment depth. *Chloroflexota* was positively associated with the presence of contaminants.

Figure 6b displays the relative abundance distribution of sediment bacterial communities at the family level. No dominant family (relative abundance >5%) was observed in this study, the top ten most abundant families were selected for analysis. The results showed that the family *CSP1-4* was the most abundant across all treatment groups (relative abundance 2.9–3.3%), followed by *Gemmatimonadaceae* (2.6–3.3%), *Casimicrobiaceae* (2.6–3.1%), *Burkholderiaceae_A* (1.3–2.4%), and *MBNT15* (2.0–3.3%). Among these, *Burkholderiaceae* has been reported to degrade various chlorophenol compounds [46,47], including 2,4,5-trichlorophenol, 2,4,6-trichlorophenol, 2,4,5,6-tetrachlorophenol, and pentachlorophenol [48]. *Casimicrobiaceae*, as a core functional group in activated-sludge systems, represents a key taxon in the microbial network of wastewater treatment plants and plays a significant regulatory role in the degradation of organic pollutants such as TCP [49]. Tubificid worms promoted an increase in the abundance of *Burkholderiaceae* in surface sediments of the H^+^ group, with increments of 1.0% compared to deeper sediments, 0.9% compared to surface sediments of the H^−^ group, and 0.7% compared to the CK^+^ group. Similarly, the abundance of *Casimicrobiaceae* increased under worm influence, with increases of 0.3% and 0.2% in surface and deeper sediments of the H^+^ group, respectively, relative to the H^−^ group. To further confirm the differences in these two functional families between the H^+^ and H^−^ groups, *t*-tests were conducted (Figure S7). The results indicated that tubificid worms significantly promoted the growth of *Casimicrobiaceae* in surface sediments and *Burkholderiaceae* in both surface and deeper sediments.

**Figure 7:**
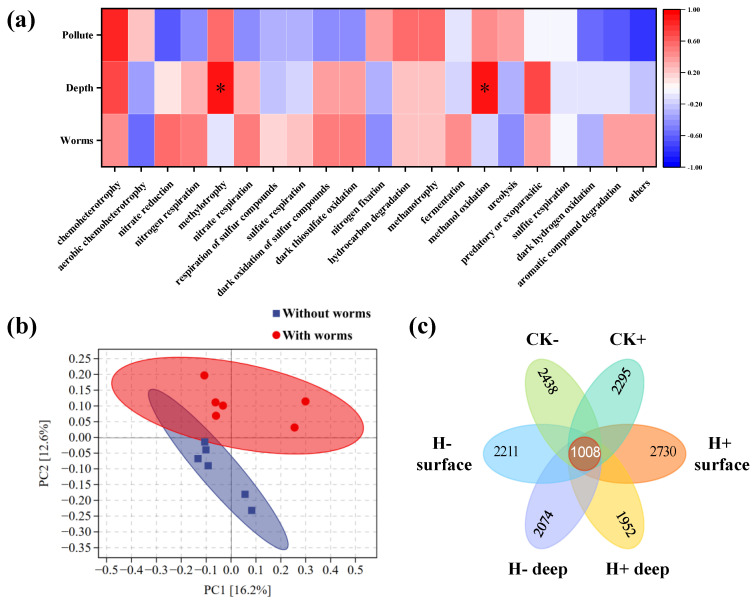
Bacterial communities in sediments. (**a**) Pearson correlation analysis with respect to pollutant concentration (0 mg/L and 5.20 mg/L), sediment depth (0–2 cm, 2–4 cm), and the presence or absence of tubificid worms (* Significant correlation at the 0.05 level). (**b**) PCoA based on OTUs showing samples with and without tubificid worms (95% confidence ellipses). (**c**) Venn diagram showing shared and unique OTUs among different groups.

Figure 6c shows the relative abundances of predicted bacterial functional groups. Chemoheterotrophy constituted the most abundant functional category across all sediment bacterial communities (27.5–30.2%). The presence of tubificid worms moderately enhanced the abundance of aerobic bacteria in sediments, with increases of 1.4% in surface sediments, 2.0% in deeper sediments, and 3.3% in the control group compared to worm-free groups. However, these differences were not statistically significant (*p* > 0.05). This observation aligns with previous findings that bioturbation by tubificid worms does not elevate sediment dissolved-oxygen levels sufficiently to support substantial proliferation of aerobic microorganisms [10]. To further elucidate the relationships between predicted bacterial functions and environmental factors, Pearson correlation analysis was performed between functional groups and system variables (with/without tubificid worms, sediment depth, presence/absence of contaminant); the resulting heatmap is presented in Figure 7a. The analysis revealed that methylotrophy and methanol oxidation exhibited significant positive correlations with sediment depth (*p* > 0.05), indicating higher relative abundances of bacteria harboring these functions in deeper sediments. Additionally, chemoheterotrophy showed a positive correlation with TCP contamination, while systems with/without worms displayed minimal correlation with most functional categories.

To compare the structure of sediment bacterial communities in the presence and absence of tubificid worms, principal coordinate analysis (PCoA) was performed based on Bray–Curtis dissimilarity, with 95% confidence ellipses drawn. As shown in Figure 7b, the bacterial communities formed two distinctly separated clusters according to the presence or absence of worms. The two principal coordinates explained 28.4% of the total variance in bacterial community structure, suggesting that the presence of tubificid worms may have induced significant overall shifts in community composition, even though differences at the phylum level were not statistically significant. Figure 7c displays a Venn diagram based on operational taxonomic units (OTUs). The analysis revealed 1008 shared OTUs across all systems, with the highest number of unique OTUs observed in the surface sediments of the H^+^ group. Additionally, the number of OTUs was consistently lower in deeper sediment layers compared to surface layers. In the group with worms and TCP, OTU richness increased relative to the control groups, whereas a decrease was observed in the group without worms. This pattern may be attributed to alterations in sediment oxygen availability and nutrient supply driven by bioturbation, which could enhance microbial metabolism and proliferation, promote synergistic growth among different bacterial groups, and ultimately lead to increased community species richness.

In summary, bioturbation by tubificid worms altered the microbial community structure in sediments, though the magnitude of change in sediment bacterial communities was considerably smaller compared to that in the overlying water. This may be attributed to the relatively limited pore space in sediments and restricted diffusion between the overlying water and the sediment matrix. The bioturbation activity of tubificid worms primarily influences the sediment–water interface, while its effects on physical mixing and oxygen transport into deeper sediment layers are limited, resulting in a weaker response from deep-sediment microbial communities. Nevertheless, analysis of the overall community structure indicates that tubificid worms significantly reshaped the bacterial community in sediments, enhancing species richness and, more importantly, enriching key functional groups involved in chlorophenol degradation, such as *Burkholderiaceae* and *Casimicrobiaceae*. This regulation of microbial community structure and function represents one of the key intrinsic mechanisms through which tubificid worms promote TCP degradation in sediments. It should be noted that the bacterial community data reflect the steady-state structure at the end of the experiment, and further investigation is needed to elucidate the dynamic processes underlying these changes.

### 3.4. Degradation Mechanism of TCP in the Presence of Tubificid Worms

#### 3.4.1. TCP Degradation Process

The TCP degradation products detected by HPLC-MS are detailed in Figure S8 and Text S8, the main intermediates identified include 4-chlorophenol (4-CP)/2-chlorophenol (2-CP), 1,2,3,5-tetramethylbenzene, and toluene. It is inferred that the key reaction steps during the degradation of 2,4,6-TCP involve: (i) hydroxylation of the benzene ring, and (ii) substitution of chlorine by OH groups, which aligns with previous research findings [50,51]. Due to the low and complex pollutant concentrations in this simulated natural environment, some intermediate products may not have been detected.

#### 3.4.2. Key Processes and Mechanisms of Enhanced TCP Degradation

Based on the above results and discussions, a high-probability mechanistic model for the promotion of TCP degradation by tubificid worms is proposed in this study, as follows.

Based on the phase distribution and depth distribution patterns of TCP, it is speculated that the degradation of TCP mainly occurs in the overlying water. As shown in Figure 4, during the early experimental period (0–14 days) when degradation was low, most of the TCP lost from the sediment was attributed to its diffusion into the overlying water. Given the direct contact between surface sediment (0–2 cm) and the overlying water, TCP in this layer diffuses more readily into the water, whereas the exchange of substances between deeper sediment layers and the overlying water is relatively restricted [16]. This aligns well with the results presented in Figure 1, which shows a pronounced decrease in TCP content in surface sediment and a comparatively smaller reduction in deeper sediment layers. This suggests that the decrease in sediment TCP is mainly due to diffusion into the overlying water, while degradation within the sediment itself is limited. Furthermore, the variation trend of ORP in the overlying water (Figure S1) indicates that during the phase of substantial TCP degradation (days 7–21), ORP decreased significantly, implying that bacterial degradation of TCP in the overlying water consumed substantial amounts of oxygen and other oxidizing substances [52,53]. In the later stage of the experiment (days 21–28), as TCP concentration in the overlying water gradually declined (Figure 3), the degradation rate slowed and ORP gradually recovered. In summary, the removal of TCP from sediment is mainly achieved through the migration of TCP from the surface layer (0–2 cm) into the overlying water, where degradation is completed, while in situ degradation within the sediment contributes only minimally.

The coupling effect of tubificid worms accelerating TCP release from sediments to the overlying water and regulating the bacterial community structure in the overlying water to enhance degradation capacity may be the most critical driving factor for tubificid worms to significantly reduce TCP content in sediments. Previous studies have confirmed that bioturbation by tubificid worms can significantly accelerate the migration of pollutants from sediments into the water column by increasing sediment porosity, promoting particle resuspension, and facilitating feeding and excretion processes [54,55]. In this study, the marked increase in overlying water turbidity (Figure S2) visually reflected the mixing process that facilitated TCP release from sediments, and it is highly consistent with the pronounced decrease in TCP content observed in surface sediments (0–2 cm). However, unlike earlier studies that solely emphasized pollutant release, this work demonstrates that the TCP concentration in the overlying water of systems with tubificid worms was significantly lower than systems without tubificid worms. This phenomenon suggests that not only does tubificid worms accelerate the release of pollutants, but, more crucially, it may enhance the overlying water’s capacity to degrade TCP by regulating the structure of bacterial communities. This hypothesis is preliminarily supported by the following observations: tubificid worms significantly increases the abundance of methylotrophic, methanol-oxidizing, and hydrocarbon-degrading bacteria in the overlying water, which are closely associated with chlorophenol degradation. Elevations in the abundance of these bacteria are generally closely linked to enhanced biodegradation of chlorophenol pollutants. In summary, tubificid worms promote the efficient removal of TCP through a synergistic mechanism. On the one hand, they accelerate the migration of TCP from sediments into the overlying water. On the other hand, they enhance the biodegradation capacity of the overlying water by modifying the bacterial community structure. Collectively, this migration–degradation coupling effect represents the most probable mechanism underlying the tubificid worm-induced reduction of TCP in sediments.

Another mechanism by which tubificid worms promote TCP removal may lie in their regulatory effects on the structure and function of the bacterial community in sediments. Although degradation may primarily occur in the overlying water, degradation in sediments also inevitably takes place, predominantly driven by bacterial metabolism. This study found that bioturbation by tubificid worms significantly altered the overall structure of the sediment bacterial community (Figure 7), mainly due to the worms improving the microenvironmental conditions of the sediment, thereby influencing bacterial community composition and metabolic activity. Specifically, worm activity elevated the redox potential of sediments (Figure S1) and enhanced the bioavailability of organic matter through their life processes. The significant decrease in TOC content in experimental groups (Figure S3) visually reflects increased consumption of organic matter by the bacterial community, further supporting the enhancement of bacterial abundance and activity [56,57]. Beyond driving changes in community structure, bioturbation by tubificid worms also significantly increased the abundance of key functional groups closely associated with chlorophenol degradation, such as *Casimicrobiaceae* and *Burkholderiaceae*, thereby directly strengthening the sediment’s capacity to degrade TCP [58]. Additionally, the biotransformation activity of the worms themselves may contribute to TCP degradation. Feeding activities of benthic organisms can secrete digestive surfactants that enhance the biotransformation of hydrophobic organic pollutants, and some bioturbators can accelerate chlorophenol degradation through enzyme-induction effects [59]. In summary, by improving the sediment microenvironment, regulating bacterial community structure, and enhancing metabolic activity, tubificid worms promoted the degradation of TCP in sediments.

**Figure 8:**
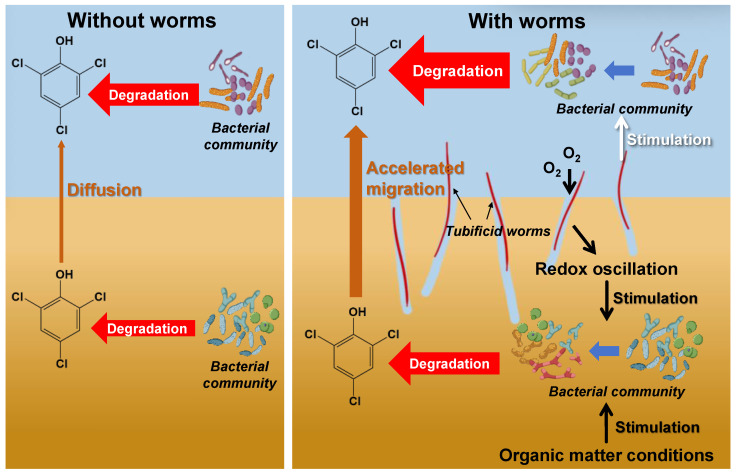
Schematic diagram illustrating the comparative mechanisms of TCP migration and degradation in systems with and without tubificid worms.

A schematic diagram illustrating the mechanisms of TCP migration and degradation is presented in Figure 8. In the system without tubificid worms, TCP in the sediment was mainly diffused into the overlying water and subsequently degraded, with only a minor fraction being degraded within the sediment itself. In the system with tubificid worms, the migration of TCP from sediment to the overlying water was significantly accelerated. Simultaneously, tubificid worms modulated the bacterial community structure in the overlying water, enhancing its degradation capacity and thereby promoting TCP removal. In addition, tubificid worms facilitated TCP degradation in the sediment by altering redox conditions, improving the availability of organic matter, and regulating the sediment bacterial community structure.

### 3.5. Environmental Implications

As key engineer of aquatic ecosystems, benthic bioturbators such as tubificid worms play an indispensable role in driving material cycling at the sediment–overlying water interface. For persistent and bioresistant refractory pollutants like chlorophenols, their environmental fate depends not only on their inherent chemical stability but is also governed by their migration and transformation processes within environmental media [35]. Previous studies have predominantly focused on the biodegradation processes of chlorophenols, with insufficient attention paid to the regulatory roles of bioturbating organisms [60,61]. Research on tubificid worms bioturbation, while generally acknowledging its capacity to accelerate pollutant release or enhance the degradation of organic contaminants by influencing sediment microbial activity, has rarely addressed its core mechanistic underpinnings [54,62]. The bioturbation process facilitated by tubificid worms converts originally immobilized and microbially inaccessible pollutants into bioavailable forms, while simultaneously stimulating shifts in bacterial communities in both overlying water and sediments, thereby enhancing pollutant removal. Therefore, in assessing sediment pollution risks, merely considering static pollutant concentrations or short-term release fluxes while neglecting the synergistic “migration–degradation” regulation mediated by benthic organisms would severely underestimate the pollutant degradation potential and the natural self-purification capacity of aquatic environments under bioturbation conditions [63]. The findings of this study provide a more comprehensive and objective summary of the synergistic regulatory mechanisms by which tubificid worms influence chlorophenol migration and degradation in the sediment–overlying water cross-phase system. This insight also provides a novel perspective for the remediation of polluted water bodies and sediments, offering a scientific basis for utilizing benthic bioturbators to enhance the self-purification capacity of aquatic environments.

### 3.6. Study Limitations and Future Research Directions

This study has certain limitations. As a laboratory microcosm simulation system was employed to focus on the single effect of tubificid bioturbation, the complex environmental factors of natural freshwater ecosystems were reasonably simplified, and the complex influencing factors present in natural systems were not considered. Therefore, extrapolation of the results to natural water bodies requires consideration of specific environmental characteristics. Additionally, this study did not directly measure the mineralization efficiency of TCP, making it impossible to accurately distinguish the relative contributions of phase transfer and biodegradation to TCP removal. Furthermore, the potential influence of symbiotic microorganisms associated with tubificid worms was not excluded, making it difficult to more precisely differentiate the coupled effects of the bioturbation behavior of tubificid worms themselves and microbial action.

Based on the above limitations, several directions for in-depth research can be pursued in the future. First, in situ field mesocosm experiments incorporating key environmental factors and biological groups from natural water bodies can be conducted to validate the applicability of the findings under natural conditions. Second, stable isotope tracing using ^13^C-TCP, combined with relevant quantitative experiments, can be employed to accurately analyze the mineralization rate, phase transfer flux, and degradation rate of TCP in the aqueous phase, thereby clarifying the contribution of each process to TCP removal. Third, by culturing and using sterile tubificid worms, multiple sets of control experiments can be designed to completely eliminate microbial interference, allowing for an in-depth analysis of the independent effects and coupling mechanisms of tubificid bioturbation and microbial action. These efforts would provide a more precise theoretical basis and technical support for the ecological remediation of freshwater sediments contaminated with chlorophenols.

## 4. Conclusions

Tubificid worms enhanced the removal of TCP from the sediment. Their influence was more pronounced in the surface sediment layer (0–2 cm) than in the deeper layer (2–6 cm). In the surface layer, the TCP removal rate increased by up to 64.1% in the presence of tubificid worms compared to the group without worms, whereas differences in removal rates among groups were smaller in the deeper layer. Phase distribution characteristics indicated that tubificid worms significantly reduced the TCP concentration in the overlying water and increased the overall degradation amount. Tubificid worms increased the turbidity and decreased the pH of the overlying water, while also raising the ORP, improving organic matter conditions in the sediment, and altering the bacterial community structure in both the overlying water and sediment. Specifically, tubificid worms promoted a significant increase in the abundance of functionally important bacterial groups in the overlying water, including those capable of chemoheterotrophy, methylotrophy, methanol oxidation, and hydrocarbon degradation. In the sediment, the families *Burkholderiaceae* and *Casimicrobiaceae* showed marked increases. TCP degradation mainly occurs in the overlying water. Tubificid worms facilitated this process by accelerating the migration of TCP from sediment to the overlying water and simultaneously modifying the bacterial community structure in the overlying water, thereby enhancing its chlorophenol degradation capacity. Secondly, the worms influenced TCP degradation in the sediment by improving the sediment microenvironment and regulating the composition and function of sediment bacteria.

## Data Availability

The original contributions presented in this study are included in the article/Supplementary Materials. Further inquiries can be directed to the corresponding author.

## Supplementary Materials

The following supporting information can be downloaded at: https://www.mdpi.com/article/10.3390/toxics14040314/s1, Figure S1: Variations in overlying water ORP across different systems; Figure S2: Variations in sediment ORP with depth across different systems; Figure S3: Variations in overlying water turbidity across different systems; Figure S4: Variations in pH of overlying water across different systems; Figure S5: Total organic carbon (TOC) content in the surface (0–2 cm) and deep (2–4 cm) layers of sediment across different systems; Figure S6. Pearson correlation analysis of sediment bacterial communities at the phylum level with respect to pollutant concentration (0 mg/L, 5.20 mg/L), sediment depth (0–2 cm, 2–4 cm), and the presence/absence of tubificid worms (* Significant correlation at the 0.05 level); Figure S7: Comparative t-test analysis of the relative abundances of Burkholderiaceae and Casimicrobiaceae across different treatment systems; Figure S8: Mass spectra of TCP and its intermediate products. References [64,65,66,67,68,69,70,71] are cited in the Supplementary Materials.
