# Supplementary material for: Environmental Behavior of 2,4,6-Trichlorophenol in the Sediment-Overlying Water System with the Presence of Tubificid Worms
Source: Toxics. 2026 Apr 7;14(4):314. doi: 10.3390/toxics14040314 (PMC13120665; doi:10.3390/toxics14040314)
Supplement: Supplementary file 1 [file toxics-14-00314-s001.zip › toxics-4196521-supplementary.pdf]

Supplementary Materials

Environmental Behavior of 2,4,6-trichlorophenol in the Sediment-Overlying Water System with the Presence of Tubificid Worms

Leyuan Zhang, Deming Dong, Xinyan Fu, Yu Zhao, Meihan Bao, Xiuyi Hua*, Dapeng Liang, Haiyang Liu

Key Lab of Groundwater Resources and Environment of Ministry of Education, Key Lab of Water Resources and Aquatic Environment of Jilin Province, College of New Energy and Environment, Jilin University, Changchun 130012, China

* Corresponding author

E-mail address: huaxy@jlu.edu.cn (Xiuyi. H)

Text S1. Analysis of TCP concentrations in sediment and overlying water

TCP extraction from sediment: The water content of different sediment samples was first determined using the oven-drying method. Subsequently, a 5 g aliquot (dry weight equivalent) of each sediment sample was weighed and placed into a beaker. Twenty milliliters (20 mL) of methanol were added for extraction over a period of 1 hour. The extract was then filtered and stored for subsequent analysis. At the beginning of the experiment, a spiking recovery test was conducted on the test sediments. TCP standard solution at a concentration of 100 mg/L was added to achieve spiking levels of 2 mg/kg and 5 mg/kg, with three parallel replicates for each spiking group. The results showed that the spiking recoveries of the samples ranged from 85.3% to 98.0%, with relative standard deviations of 3.3% to 8.6% (<30%), indicating good reproducibility.

Separation and enrichment of TCP from overlying water and its particulates: One hundred milliliters (100 mL) of overlying water were collected and subjected to vacuum filtration to separate the aqueous phase from the filter paper retaining the suspended particulate matter. The filter paper was air-dried, and the particulates were extracted by ultrasonication with 30 mL of a dichloromethane and n-hexane mixture (2:1, v/v). The extract was transferred to a separatory funnel. A volume of water equal to twice that of the extract was added, and the pH was adjusted to be greater than 12 using 5 mol/L NaOH. The mixture was shaken vigorously, allowed to settle, and the lower organic phase was discarded. The retained alkaline aqueous phase was then acidified to pH < 2 using 3 mol/L HCl. Both the original overlying water and this acidified aqueous solution were separately subjected to solid-phase extraction using HLB cartridges. The eluate from each was collected, evaporated, and concentrated to 0.5 mL. Finally, each concentrate was diluted to a final volume of 1.0 mL with purified water for analysis. For each batch of samples, blank controls (ultrapure water subjected to the same extraction procedure) and spiked recovery samples (ultrapure water or blank filter membranes spiked with known concentrations of TCP standard solution and extracted using the same procedure) were concurrently prepared. The TCP spiking recoveries were maintained within the range of 85.8% to 115.0%, with relative standard deviations below 10%, meeting the accuracy requirements for the extraction of trace organic pollutants.

TCP concentration analysis: The concentration of TCP was analyzed using High-Performance Liquid Chromatography (HPLC, LC-20AB, Shimadzu). The HPLC system was

equipped with a UV detector and a 5 μm C18 column (4.6 × 250 mm). The mobile phase consisted of a mixture of ultrapure water and methanol (v/v = 20:80), delivered at a flow rate of 0.8 mL/min. The detection wavelength was set at 296 nm, the column temperature was maintained at 35 °C, and the injection volume was 20 μL.

Text S2. Bacterial community 16S rRNA gene sequencing

The detailed methodology was as follows: Total genomic DNA was extracted from 0.25 g of sediment samples using the MagBeads FastDNA Kit for Soil (116564384, MP Biomedicals). DNA concentration was quantified using a NanoDrop NC 2000 spectrophotometer (Thermo Fisher Scientific, Waltham, MA, USA), and DNA quality was assessed by 0.8% agarose gel electrophoresis. The V3–V4 hypervariable region of the bacterial 16S rRNA gene was amplified via polymerase chain reaction (PCR) using the barcoded universal primers 338F (5'-barcode + ACTCCTACGGGAGGCAGCA-3') and 806R (5'-barcode + GGACTACHVGGGTWTCTAAT-3'). The PCR products were verified by 2% agarose gel electrophoresis. Subsequently, the amplified libraries were sequenced on an Illumina NovaSeq high-throughput sequencing platform for downstream analysis.

Text S3. Variations in overlying water oxidation reduction potential

The variation in oxidation-reduction potential (ORP) of the overlying water is illustrated in Figure S1. The group without tubificid worms exhibited relatively minor fluctuations, decreasing from an initial value of 509.4 ± 3.1 mV to a minimum of 475.4 ± 5.3 mV. In contrast, the ORP in TCP-contaminated experimental groups declined rapidly after day 7, with each decreasing by over 160 mV. The significant decrease in ORP within the experimental groups is attributed to the substantial consumption of dissolved oxygen, as well as oxidizing substances such as nitrate and Fe^{3+}, during the degradation of contaminants (TCP and the co-solvent methanol). Concurrently, the accumulation of reductive metabolic byproducts, including CO_2 and NH_4^+, generated through microbial respiration, further contributed to the sharp decline in system ORP.

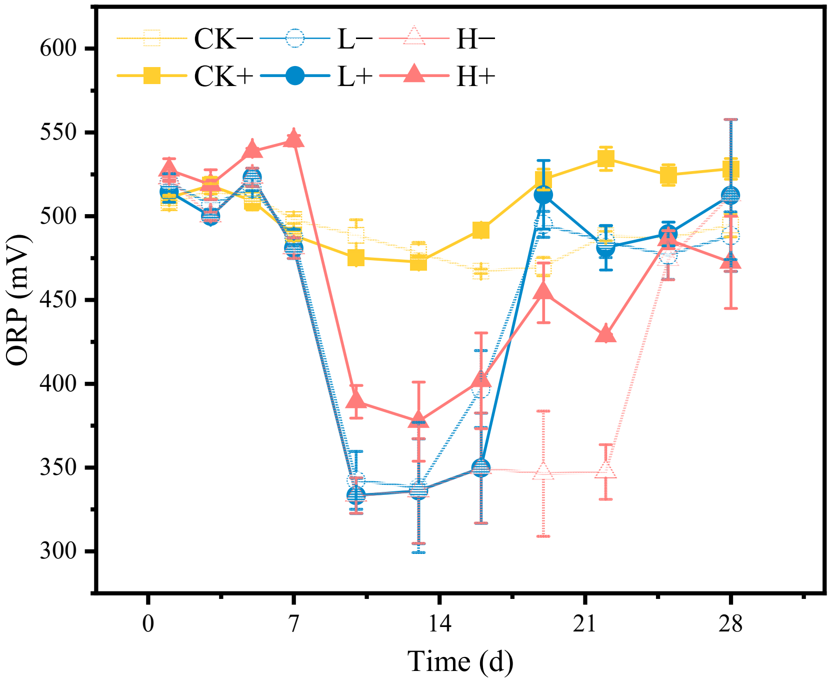

Figure S1. Variations in overlying water ORP across different systems.

Text S4. Sediment oxidation-reduction potential

The sediment oxidation-reduction potential (ORP) is presented in Figure S2. ORP values decreased progressively with increasing sediment depth. Sediments containing tubificid worms exhibited consistently higher ORP compared to those without worms. This increase can be attributed to bioturbation by tubificid worms at the water–sediment interface, which promotes the infiltration of overlying water. Given that the overlying water typically possesses a higher ORP than the sediment, this process elevates sediment ORP levels. Furthermore, through feeding and burrowing activities, tubificid worms enhances sediment porosity and aeration, leading to increased oxygen penetration depth and a significant rise in sediment ORP (Yan et al., 2020; Ridall et al., 2024). In addition, ORP values were lower in TCP-contaminated experimental groups than in the control group, although variations in TCP concentration showed limited influence on ORP. This reduction is likely due to the microbial degradation of TCP and the co-solvent methanol in sediments, which consumes oxygen and other electron acceptors (e.g., nitrate and sulfate), thereby lowering the redox potential. Since methanol was present in substantially higher concentrations than TCP in this experiment, it likely played a dominant role in reducing ORP in the contaminated treatments, resulting in a non-linear relationship between TCP concentration and ORP. While increased sediment ORP may directly affect TCP behavior, its primary influence is expected to be on the composition and metabolic

activity of sediment bacterial communities.

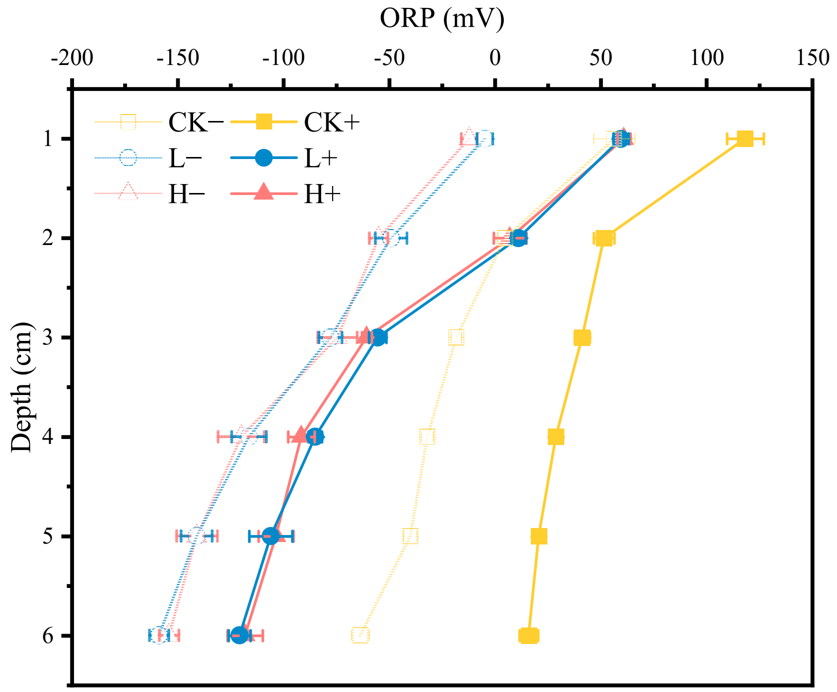

Figure S2. Variations in sediment ORP with depth across different systems.

Text S5. Variations in overlying water turbidity

Variations in the turbidity of the overlying water are depicted in Figure S3. In the experimental groups containing tubificid worms, turbidity exhibited a sharp initial rise, followed by a decline and eventual stabilization. This pattern is attributed to increased sediment porosity and continuous bioturbation by tubificid worms at the sediment surface, which promotes particle resuspension and consequently leads to a rapid increase in turbidity. In the experimental groups without tubificid worms, turbidity gradually increased after the seventh day of the experiment, likely due to gas production during microbial degradation of the contaminants (TCP and the co-solvent methanol). Bubble formation and upward migration through the overlying water likely facilitated the transport of particles, which were further mobilized by electrostatic interactions, enhancing their movement into the water column. Furthermore, turbidity in the experimental groups decreased relative to the control after day 7. This reduction is likely a consequence of decreased tubificid worms activity under TCP-induced toxicity, leading to diminished sediment disturbance and a subsequent gradual decline in turbidity.

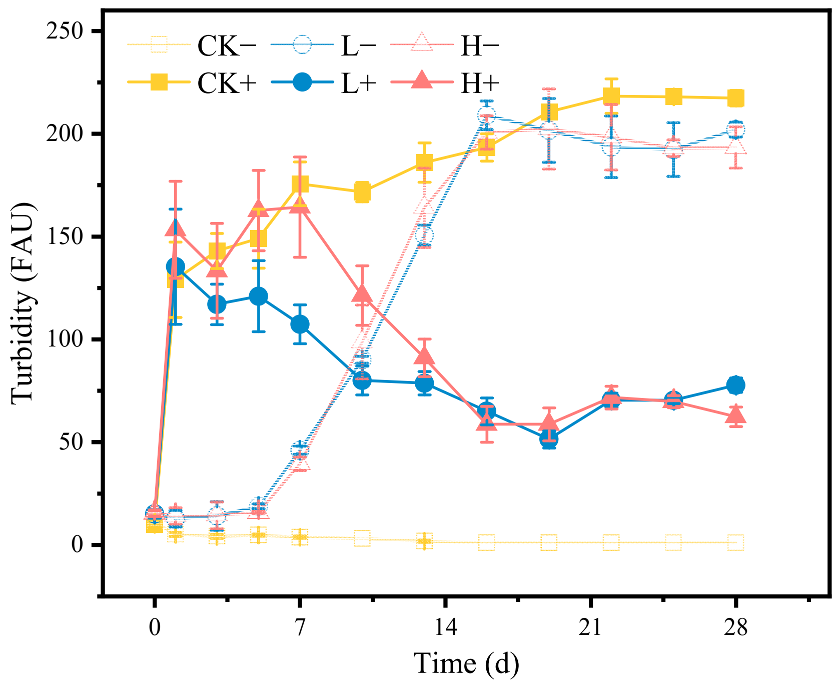

Figure S3. Variations in overlying water turbidity across different systems.

Text S6. Variation in pH of overlying water

The pH variation in the overlying water is presented in Figure S4. Systems without tubificid worms maintained a stable pH within the range of 7.02–7.36 without significant fluctuations. In contrast, systems containing tubificid worms consistently exhibited lower pH values compared to those without worms, showing a gradual decline followed by stabilization. Among these, experimental groups with tubificid worms had lower pH values than their corresponding control groups, and higher TCP concentrations corresponded to lower pH levels. The pH reduction induced by tubificid worms activity results from multiple contributing factors. First, increased excretion and respiration by tubificid worms elevate CO_2 (H_2CO_3) levels. Dissolved CO_2 significantly raises hydrogen ion (H^+) concentrations through the carbonate equilibrium system (Zhu et al., 2006). Second, mucus secretion and metabolic excretion from tubificid worms introduce substantial acidic substances, including organic acids such as pyruvate and lactate generated from amino acid metabolism (F. Riemann, 1988), as well as sulfates formed via oxidation of sulfur-containing compounds ((Jonas Martin Svensson, 2001). Third, bioturbation by tubificid worms may enhance nitrifying bacterial activity in sediments, further contributing to pH reduction. Additionally, higher TCP concentrations were associated with faster pH decline, likely due to the activation of specific microbial acidogenic metabolic pathways during contaminant degradation.

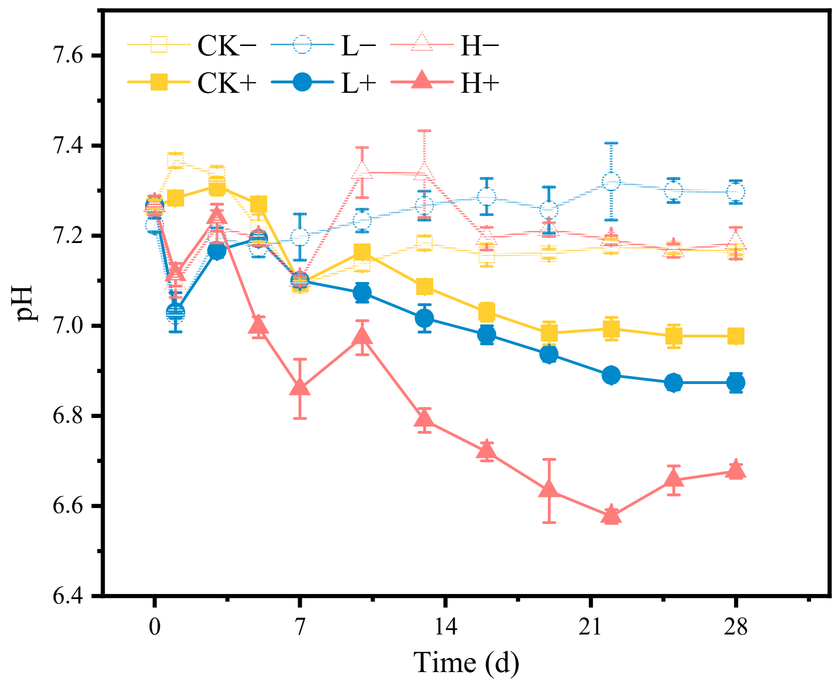

Figure S4. Variations in pH of overlying water across different systems.

Text S7. Total organic carbon (TOC) content in sediment

The TOC content in the surface (0–2 cm) and subsurface (2–4 cm) layers of sediment across different systems is shown in Figure S5. Prior to analysis, live tubificid worms and a small number of carcasses were removed during sediment sampling. As TOC measurement was conducted on day 28, the influence of contaminants (TCP and co-solvent methanol) on TOC results is considered minimal. Sediment TOC content across all groups ranged from 1.7% to 2.2%. In the control group, the presence of tubificid worms increased sediment TOC, with increments of 0.15% in the surface layer and 0.064% in the subsurface layer. Conversely, in the experimental groups, the presence of tubificid worms reduced sediment TOC content—by 0.18% and 0.20% in the high-concentration group, and by 0.10% and 0.12% in the low-concentration group—for surface and subsurface layers, respectively. No significant differences in sediment TOC were observed among the three groups without tubificid worms. Changes in sediment TOC content result from a combination of complex factors. On one hand, tubificid worms contribute to TOC input through their biomass and excretory products, increasing TOC levels. On the other hand, tubificid worms ingest substantial amounts of sediment, consuming organic detritus, bacteria, and algae, thereby reducing TOC content. Within the gut, these organic materials are partially digested and decomposed through enzymatic and symbiotic microbial activities, resulting in the excretion of organic matter more readily utilized by microorganisms.

This process substantially increases the surface area for microbe–organic matter interaction, significantly accelerating the microbial mineralization of organic carbon and ultimately leading to a reduction in sediment TOC. Moreover, under the influence of tubificid worms, both the abundance and activity of sediment microorganisms are enhanced, further promoting TOC depletion (Shen et al., 2017; Yang et al., 2020). In co-metabolic systems, readily degradable substrates can serve as energy and carbon sources for microbial growth, facilitating the degradation of phenolic compounds (Wang and Sun, 2020). Therefore, alterations in organic matter conditions within sediments may also affect TCP degradation dynamics.

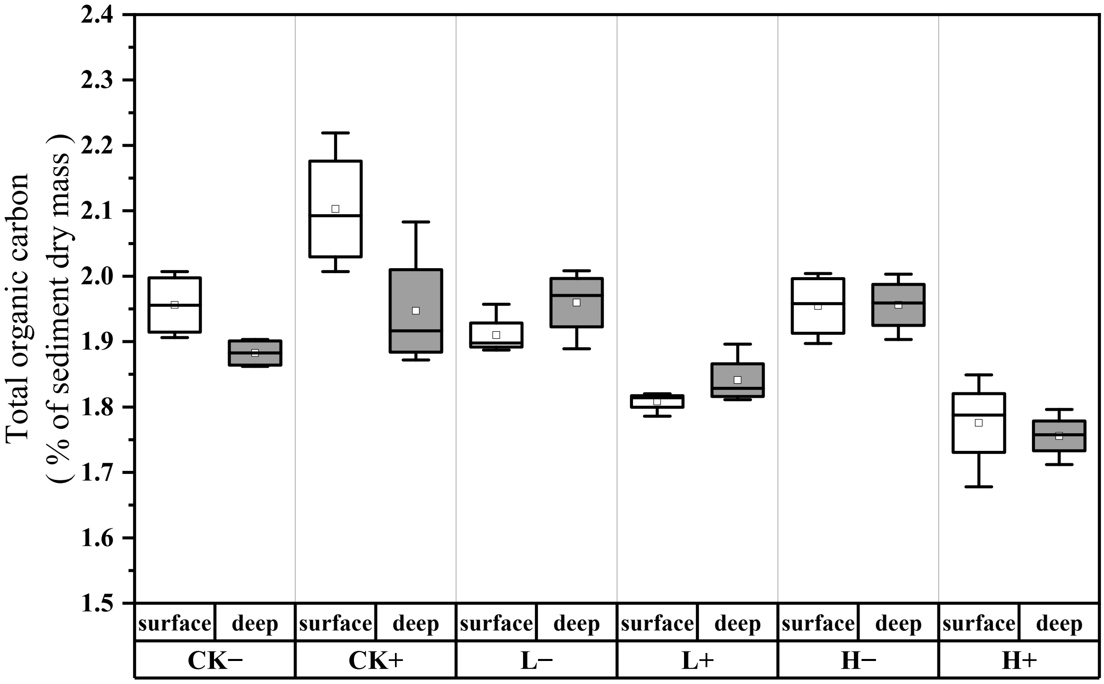

Figure S5. Total organic carbon (TOC) content in the surface (0–2 cm) and deep (2–4 cm) layers of sediment across different systems.

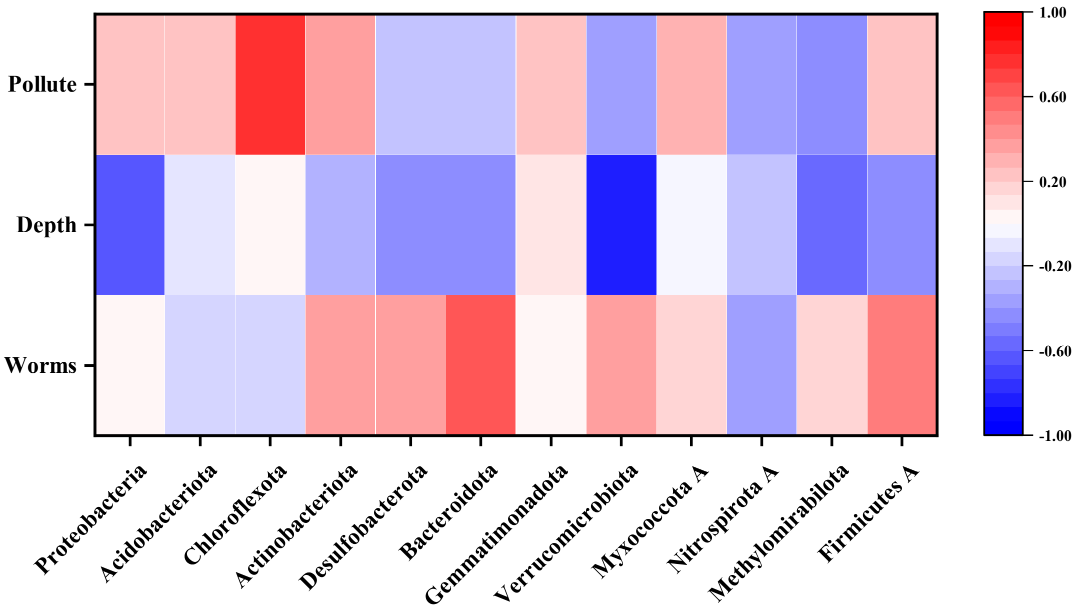

Figure S6. Pearson correlation analysis of sediment bacterial communities at the phylum level with respect to pollutant concentration (0 mg/L, 5.20 mg/L), sediment depth (0-2 cm, 2-4 cm), and the presence/absence of tubificid worms (* Significant correlation at the 0.05 level).

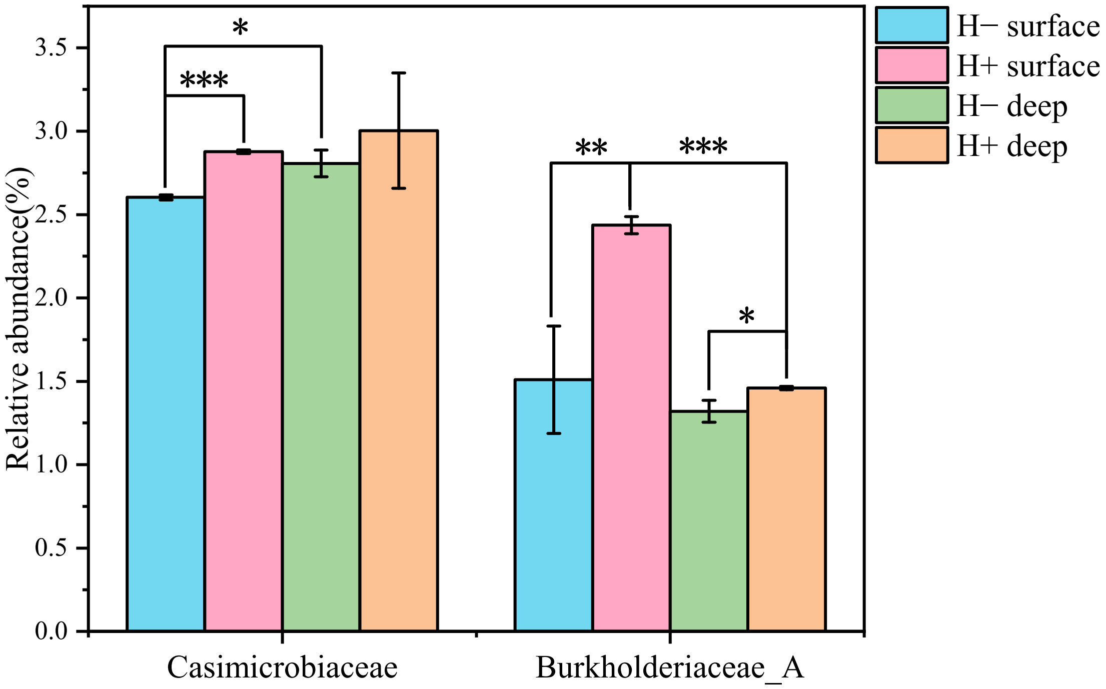

Figure S7. Comparative T-test analysis of the relative abundances of *Burkholderiaceae* and *Casimicrobiaceae* across different treatment systems.

Text S8: Identification of TCP Degradation Intermediates

HPLC-MS analysis was employed to identify potential intermediates generated during TCP degradation (Figure S9). Based on the analytical results, primary peaks were observed at retention times of 4.68 min (peak b, *m/z* = 130), 7.13 min (peak c, *m/z* = 135), 8.78 min

(peak d, *m/z* = 91), 6.18 min (peak e, *m/z* = 212), and 6.57 min (peak f, *m/z* = 233). These peaks correspond to 4-chlorophenol (4-CP) / 2-chlorophenol (2-CP), 1,2,3,5-tetramethylbenzene, toluene, and other compounds, respectively.

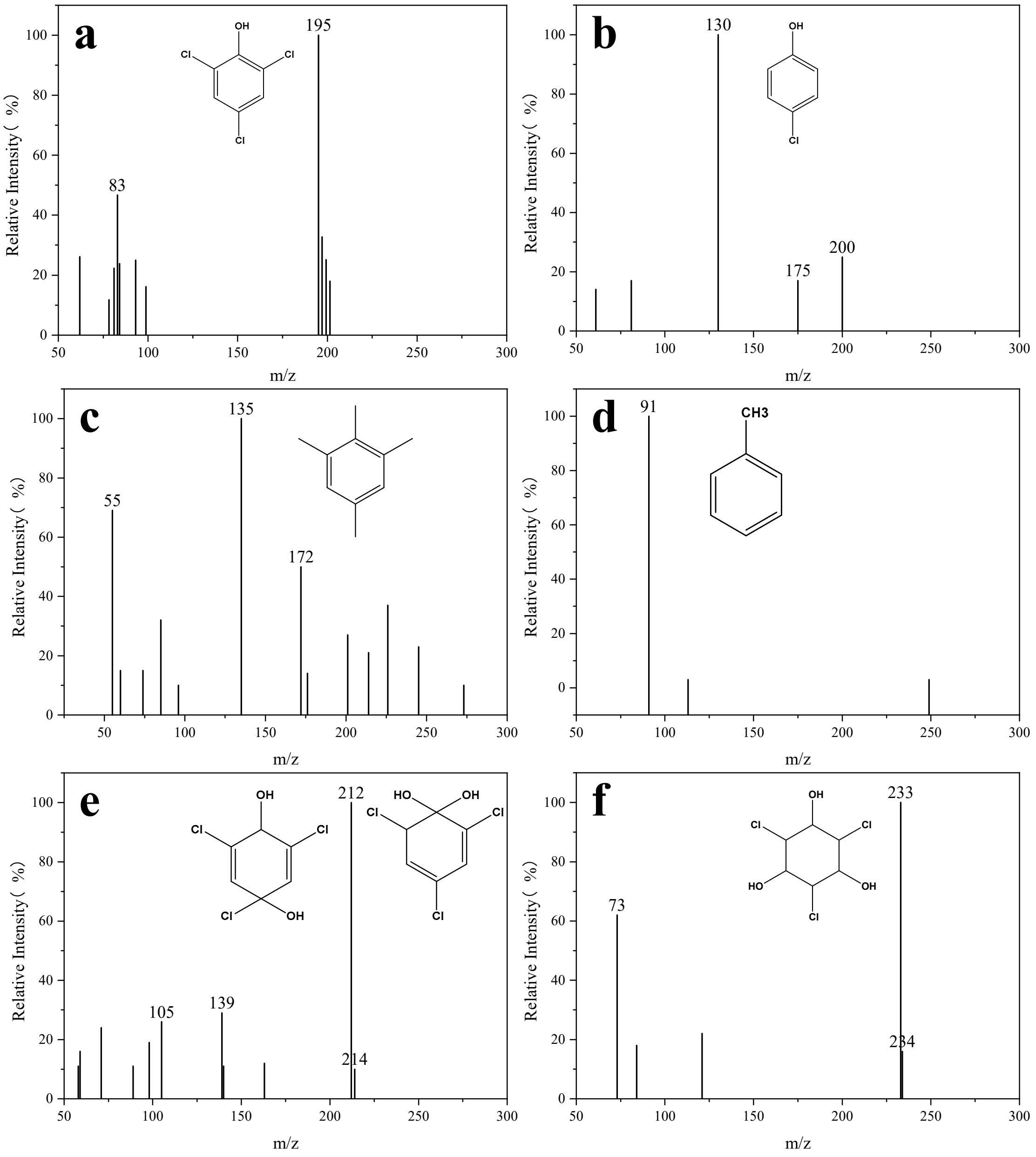

Figure S8. Mass spectra of TCP and its intermediate products.

References

Ridall, A., Maciute, A., Nascimento, F.J.A., Bonaglia, S., Ingels, J. Microplastic-induced shifts in bioturbation and oxygen penetration depth in subtidal sediments. *Mar. Pollut. Bull.* **2024**, *209*, 117074.

Yan, W., Chen, M., Liu, L., Wu, T., Zhang, Y., Wang, H., Xing, X., Fan, K. Mechanism of phosphorus mobility in sediments with larval (*Propsilocerus akamusi*) bioturbation. *Environ. Sci. Pollut. Res.* **2020**, *27*, 7538-7548.

Zhu, Q., Aller, R.C., Fan, Y. Two-dimensional pH distributions and dynamics in bioturbated marine sediments. *Geochim. Cosmochim. Ac.* **2006**, *70*, 4933-4949.

F. Riemann, M.S. Carbon dioxide as an attractant for the free-living marine nematode. *Mar. Biol.* **1988**, *98*, 81-85.

Jonas Martin Svensson, A.E.L.L. Nitrification and denitrification in a eutrophic lake sediment bioturbated by oligochaetes. *Aquat. Microb. Ecol.* **2001**, *23*, 177-186.

Yang, J., Wan, Y., Cao, Z., Zhang, M., Zheng, F., Leng, X., Zhao, D., An, S. Enhanced organic matter decomposition in sediment by *Tubifex tubifex* and its pathway. *J. Environ. Manage.* **2020**, *275*, 111281.

Shen, H., Jiang, G., Wan, X., Li, H., Qiao, Y., Thrush, S., He, P. Response of the microbial community to bioturbation by benthic macrofauna on intertidal flats. *J. Exp. Mar. Biol. Ecol.* **2017**, *488*, 44-51.

Wang, J., Sun, Z. Exploring the effects of carbon source level on the degradation of 2,4,6-trichlorophenol in the co-metabolism process. *J. Hazard. Mater.* **2020**, *392*, 122293.
